# On-Surface Synthesis within a Porphyrin Nanoring Template

**DOI:** 10.1038/s41598-019-45359-w

**Published:** 2019-06-27

**Authors:** Chris J. Judd, Dmitry V. Kondratuk, Harry L. Anderson, Alex Saywell

**Affiliations:** 1 School of Physics and Astronomy, University of Nottingham, Nottingham, NG7 2RD UK; 2 Department of Chemistry, Oxford University, Oxford, OX1 3TA UK

**Keywords:** Surface chemistry, Scanning probe microscopy, Heterogeneous catalysis

## Abstract

On-surface synthesis provides a route for the production of 1D and 2D covalently bonded polymeric structures. Such reactions are confined to the surface of a substrate and the catalytic properties of the substrate are often utilised to initiate the reaction. Recent studies have focused on the properties of various crystallographic planes of metallic substrates, as well as native surface features such as step-edges, in an effort to provide control over the pathway of the reaction and the resultant products. An alternative approach is to template the catalytic surface with a porous molecular overlayer; giving rise to well-defined surface regions within which an on-surface reaction may be confined. Here we present a methodology where macromolecular templates are used to confine an on-surface reaction. Cyclic porphyrin polymers, nanorings - consisting of 40 porphyrin units with internal diameter 13 nm, are used to form a template on a Au(111) surface, and an on-surface Ullmann-type coupling reaction is initiated within the nanoring template. The surface confined template and covalently coupled reaction products are investigated and characterised with scanning tunnelling microscopy.

## Introduction

The on-surface synthesis of 1D and 2D covalently bonded structures, performed on metal substrates under ultra-high vacuum (UHV) conditions, has been achieved through a variety of different strategies^1–5^: facilitating the synthesis of molecular chains^6–11^, graphene nanoribbons^12,13^, and 2D molecular frameworks^14–16^. Many studies have focused on the transfer of classical solution-phase reactions (e.g. Ullmann coupling and Glaser coupling)^2^ onto supporting substrates held under UHV conditions. Such reactions are often initiated via the interaction between the vacuum deposited monomer species and the catalytic surface, resulting in the breaking of covalent bonds (e.g. carbon-halogen bonds in Ullmann coupling) to produce a reactive intermediate species that may then participate in the on-surface formation of a covalently bonded product. Confining molecules to two dimensions in this way can lead to stereo- and regioselective reaction pathways not seen in solution synthesis (due to confinement to the quasi-2D plane provided by the supporting substrate), which in turn can lead to the selective formation of low dimensional molecular structures. Such on-surface reactions can be characterised *in-situ* by scanning probe techniques, such as scanning tunnelling microscopy (STM) and atomic force microscopy (AFM), offering insights into the structure of products and the reaction process. Ullmann-type coupling reactions have been widely investigated, and have been employed to produce a variety of products including: dimers^17^, linear chains^14,18–21^, and extended networks^16,22,23^. In addition, the sub-molecular resolution offered by scanning probe techniques has allowed on-surface reactions to be characterised at various stages (e.g. initial, intermediate, and final states)^24,25^.

Covalently bonded structures produced by implementing current on-surface synthesis protocols often exhibit a high degree of disorder, particularly over length scales greater than a few nanometers^16,26^. A promising approach is to influence, and direct, the on-surface reaction by utilising the structure of the catalytically active surface. Previous studies have focused on two main areas: (i) Native surface structures, such as step edges^10,11^, or the topography of anisotropic crystallographic planes^27,28^, have been used to allign reactant molecules in a specific geometry, assisting the formation of more ordered structures. (ii) Surface reconstructions, such as Cu(110)-(2x1)O, have been produced for use as templates; spatially confining molecules in order to influence the structure of the covalently coupled products formed via an on-surface reaction^29^. More recently, we have demonstrated the use of molecular structures as templates by employing a self-assembled extended porous network to confine molecules, facilitating an investigation of reactions within the pores^25^. In summary: A porous hydrogen-bonded supramolecular network was formed on a Ag(111) surface, with the substrate acting as a catalyst for the breaking of intramolecular carbon-halogen bonds of deposited reactant species confined within the pores. This approach has the potential to allow catalytic surfaces to be templated by molecular systems, leading to surfaces with well-defined reactive regions. However, the internal dimensions of such networks can be similar to those of the precursor molecules and hence alternative templates for the formation of extended structures are required. Some of the largest macromolecular systems synthesised to-date are porphyrin nanorings^30,31^ (cyclic structures consisting of 10 s of covalently bonded porphyrins) which offer an ideal porous material with which to template a catalytic surface.

**Figure 1:**
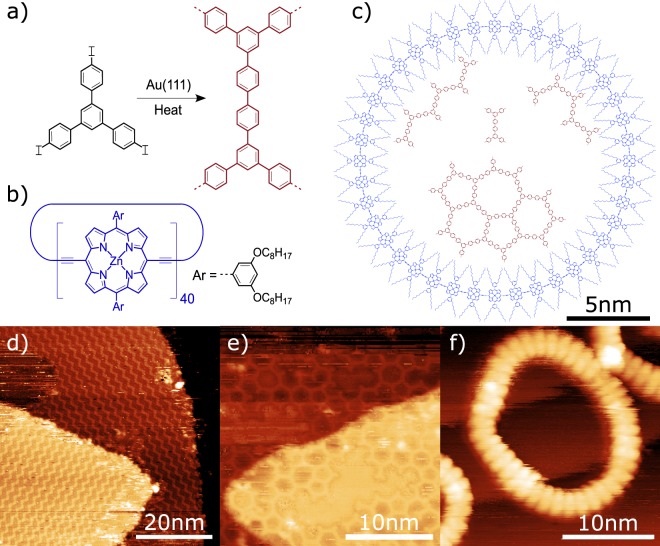
Chemical structures and STM characterisation of the TIPB and c-P40 molecular species. (**a**) Schematic diagram of the TIPB Ullmann-type coupling reaction forming covalently bonded structures. (**b**) Chemical structure of the c-P40 nanoring. (**c**) Schematic showing a c-P40 ring with examples of covalently bonded structures which may be formed via Ullmann-type coupling of TIPB inside the ring. (**d**) STM image of the self-assembled TIPB zigzag structures formed on the Au(111) surface; demonstrating that iodine has not dissociated from the molecules (*I*_*set*_ = 30 pA, *V* = −1.8 V). (**e**) STM image showing a region of the two-dimensional extended network of covalently bonded TPB formed on Au(111) following activation of the on-surface reaction (*I*_*set*_ = 50 pA, *V* = 1.8 V). (**f**) STM image of a stack of c-P40 nanorings on Ag(111) showing clear resolution of individual porphyrin units (*I*_*set*_ = 10 pA, *V* = −1.8 V).

Here we investigate, and characterise, an on-surface reaction via room temperature STM studies, performed under UHV conditions. Porphyrin nanorings are employed to act as areas of confinement for the on-surface Ullmann-type coupling reaction of 1,3,5-tris(4-iodophenyl)benzene (TIPB) on Au(111) [NB: the terminology Ullmann-type reaction is used here to refer to on-surface reactions employing Au, Ag and Cu substrates and halogen functionalised reactant molecules, as the original Ullmann reaction was defined using a Cu catalyst]. The reactant molecule, TIPB, consists of a central phenyl ring with three outer iodobenzene groups (shown in Fig. 1a) and is known to react through an Ullmann-type coupling mechanism^17,26,32^. Molecules adsorbed on the surface undergo dissociation of the carbon-halogen (C-X) groups, when sufficient thermal energy is available, and form reactive species that diffuse across the surface. When two such species meet they form a single covalent bond, resulting in the formation of polymeric structures. The dissociation of the C-I bond within the TIPB molecule leads to the formation of a 1,3,5-triphenylbenzene (TPB) monomer unit, from which the extended polymers are formed. In order to template the surface we employ a cyclic porphyrin structure^31,33^ - c-P40, shown in Fig. 1b. The ring consists of 40 Zn-centred porphyrin units, each with two 1,3-bis(octyloxy)benzene side groups, covalently bonded to form a cyclic polymer. When deposited upon a surface these rings are known to be flexible, primarily forming ellipsoidal geometries^34^, and have been observed to form stacks up to 1 nm in height (for a stack of four rings)^31^. The geometry of the rings allows molecular species to be confined within a well defined area (as previously demonstrated for C_60_)^35^; and in this work we demonstrate that the pores are of sufficient size to allow extended covalent structures to form inside of them.

As the large size of the c-P40 nanorings rings prohibits deposition via traditional UHV techniques, such as thermal sublimation, we employ an electrospray ionisation deposition technique (previously used for similar porphyrin based systems)^31,36^; allowing the molecules in solution to be transferred to a surface held under UHV. Similar approaches have been applied to investigate a range of molecules including proteins^37^, single molecule magnets^36,38^, and a variety of other species^39,40^.

A structural model for a c-P40 ring and several potential reaction products (1D, 2D, and dimer) are shown in Fig. 1c. A single c-P40 (shown in blue) is used to contain several covalently bonded TIPB structures (red). These may consist of various linear chains and 2D networks, formed through Ullmann-type coupling of TIPB monomer units deposited inside the ring. The maximum size of a covalently bonded structure that could form inside a c-P40 ring would consist of 54 TPB units and be approximately 13 nm in diameter (example shown in Supplementary Information), however, the formation of less-ordered structures consisting of a small number of TPB units is also possible.

## Methods

Experiments were performed under UHV conditions using an Omicron STM-1 system, base pressure 10^−9^ mbar. STM images were acquired in constant current mode (bias applied to sample) at room temperature using electrochemically etched tungsten tips (coated in gold during tip optimisation by controlled indentation into the surface). The substrate, a gold on mica sample (Georg Albecht PVD - GmBH), was prepared by argon ion sputtering (0.75 keV, *I*_*drain-current*_ = 1.5 *μ*A, *P*_*argon*_ ≈ 2.5 × 10^−5^ mbar) for 30 minutes, followed by annealing at ~400 °C for 25 minutes. Images of the surface acquired after the sputter-anneal cycle showed the characteristic herringbone reconstruction of the Au(111) surface. The c-P40 nanoring material was synthesised *ex-situ* using a previously published method based upon Vernier templating^30,31^. c-P40 was deposited from a solution of toluene and methanol (3:1 mixture) onto a clean Au(111) surface held at 10^−6^ mbar via electrospray deposition (similar to a previously reported procedure)^30,31^. Depositions were performed with a solution flow rate of 4 *μ*L/hour (for a period of 30 minutes), with the electrospray event initiated by applying a potential difference of ~1.8 kV between the electrospray tip and entrance capillary to the UHV system. TIPB (Sigma Aldrich) was deposited onto the surface by exposing the Au(111) surface to the flux from a Knudsen-type evaporator heated to 180 °C for 5 minutes.

## Results and Discussion

Preliminary experiments to characterise TIPB and c-P40 on Au(111), via STM, were performed; STM images are shown in Fig. 1d–f. TIPB was deposited via sublimation on to a Au(111) sample at room temperature (Fig. 1d) and was found to form a ‘zigzag’ phase, as observed previously^17^, indicating that iodine had not dissociated from the molecule (see Supplementary Information, Fig. S2). Annealing the surface to ~110 °C induced catalytic cleaving of the C-I bonds within TIPB, leading to the formation of TPB as part of the Ullmann-type coupling reaction. TPB molecules are then observed to form two-dimensional networks, shown in Fig. 1e. The structures formed exhibit a non-regular arrangement with cells of square, pentagonal, hexagonal, and higher-order polygonal symmetries^26,41^. When deposited onto a separate, clean, Au(111) surface the c-P40 rings (transferred from solution to the substrate in UHV via electrospray) were observed to form stacks on the surface, typically 2–4 units high (between 0.4 and 1.0 nm); similar to that observed in previous experiments (~0.4 nm for double stacks, ~0.7 nm for triple stacks, and ~0.1 nm for single height rings)^31^ where the number of rings in a stack can be affected by the composition of the solvent used for electrospray deposition^34^. c-P40 is known to be flexible and very few circular rings were observed, with most arranged in stacks of ovals (flattening factor *f* = 0.31 ± 0.03). Additionally, no diffusion of the rings was observed during the STM measurements with the adsorbed rings mainly present at step edges. Sub-molecular resolution STM images of rings allows features corresponding to individual porphyrin units to be resolved (Fig. 1f), providing confirmation that each ring contained 40 units (image for c-P40 on Ag(111)). The separation between each unit was found to be 1.31 ± 0.09 nm, in agreement with previous results^7,31^. Rings were found to have an average internal diameter of 13.0 ± 0.2 nm.

**Figure 2:**
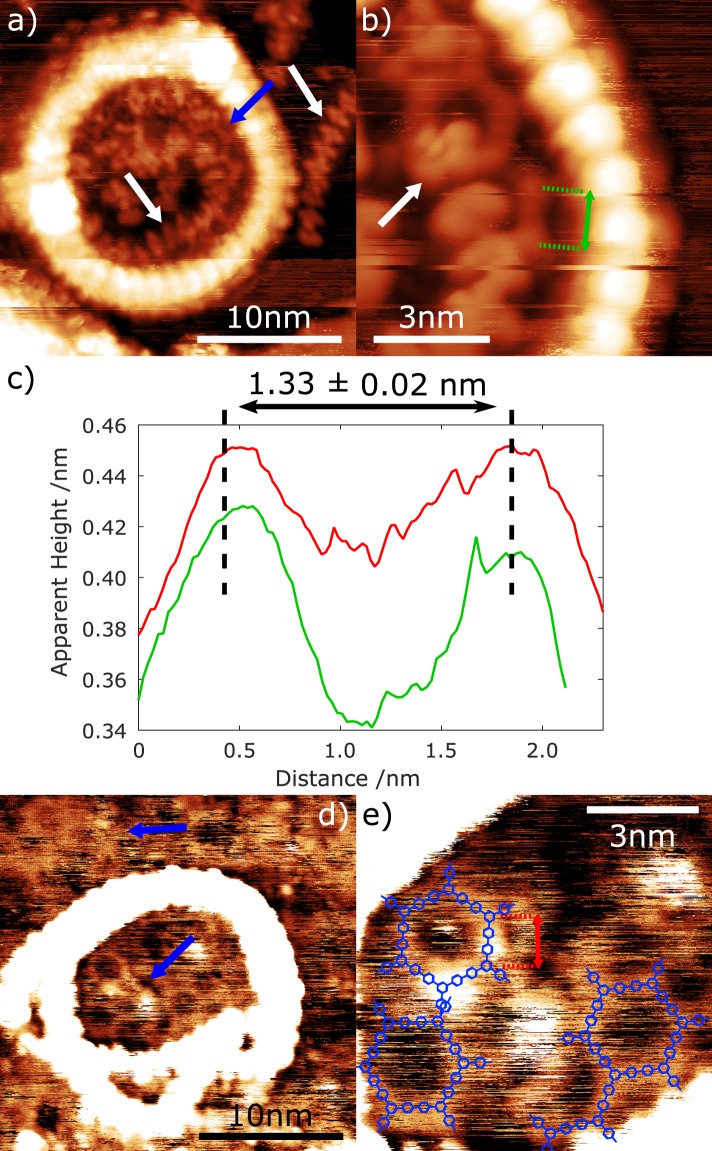
STM images acquired following the deposition of TIPB onto a Au(111) surface pre-patterned with c-P40 nanorings, followed by annealing the surface to ~110 °C. Images acquired with *I*_*set*_ = 20 pA, *V* = −1.8 V. (**a**) Image showing TPB covalently bonded structure inside c-P40 ring (blue arrow) and broken chains of porphyrins (white arrow). (**b**) Close up of (**a**) showing clear TPB covalent structure. Porphyrin chain covering structure indicated with white arrow. The size and direction of the average line profile shown in (**c**) is indicated in green. (**c**) Average of 5 line profiles showing separation of TPB covalent structures in (**b)** (green) and (**e**) (red). (**d**) TPB structures seen inside and outside of c-P40 ring indicated with blue arrows (contrast within the c-P40 structure is ‘saturated’ in the image to show surface structures). (**e**) Close up of TPB structures in (**d**). Proposed molecular structures overlaid. The size and direction of the average line profile shown in **(c)** is indicated in red.

Following the studies of the homomolecular systems, a templated Au(111) surface was prepared by first depositing c-P40 (obtaining a surface coverage of approximately 1.1 ± 0.3 c-P40 stacks per 100 nm^2^). TIPB was subsequently deposited using the methodology described above. Heating the surface to ~110 °C initiated the Ullmann-type coupling reaction between the TIPB molecules and resulted in the formation of extended TPB structures; observed at several locations on the surface, both inside and outside of the stacked c-P40 rings. An STM image of a c-P40 ring on the Au(111) surface is shown in Fig. 2a. Sections of porphyrin polymers, which are not part of the cyclic c-P40 rings, are observed on the surface, both inside and outside of the rings (indicated by white arrows in Fig. 2a) and are attributed to c-P40 species in which a covalent bond connecting neighbouring porphyrin units has been broken. It is evident from these structures that the elevated temperatures required to initiate the on-surface coupling of TIPB also lead to disruption of the c-P40 ring structures (via the breaking of covalent bonds within the nanoring).

Characterisation of the surface following annealing reveals that TPB structures are present inside the c-P40 rings (Fig. 2a - blue arrow). A close-up image acquired within this ring is shown in Fig. 2b. The structures here are attributed to a chain of covalently bonded TPB units, potentially incorporating a TPB macrocycle (partially obscured by the broken c-P40 ring - white arrow). The covalent nature of this structure is determined from measurements of the separation between vertices of the observed chain (found to be 1.33 ± 0.02 nm - position of measured dimensions shown in Fig. 2b with averaged line profile data shown in Fig. 2c in green). This measurement agrees with the expected value of 1.31 nm for four covalently bonded aryl units^26^ and suggests that the observed structures are formed from covalently bonded TPB.

Figure 2d shows a distorted stack of nanorings with several cyclic network features present both inside and outside of the ring (the contrast has been ‘enhanced’ to show details of structures within the ring, leading to a corresponding reduction in the definition of the c-P40 ring). A line profile extracted from the image is shown in Fig. 2c (red) and shows agreement with the previous measured separation between the vertices of the structure (structure in Fig. 2b); consistent with covalently bonded TPB structures. Based on the characteristic porous structures observed, and their length scales, we attribute these features to small regions of covalently bonded TPB networks. Figure 2e shows a close up of the structures inside the ring, with suggested positions for the TPB structures overlaid. Due to the mobility of material on the surface (e.g. motion of the porphyrin chain forming part of the broken ring) achieving stable imaging conditions within the stacked rings is challenging. However, it is evident from these measurements that the molecular structures observed within the cP40 rings have the expected dimensions and geometry for the coupled product formed from TIPB species reacted within the porphyrin nanorings.

**Figure 3:**
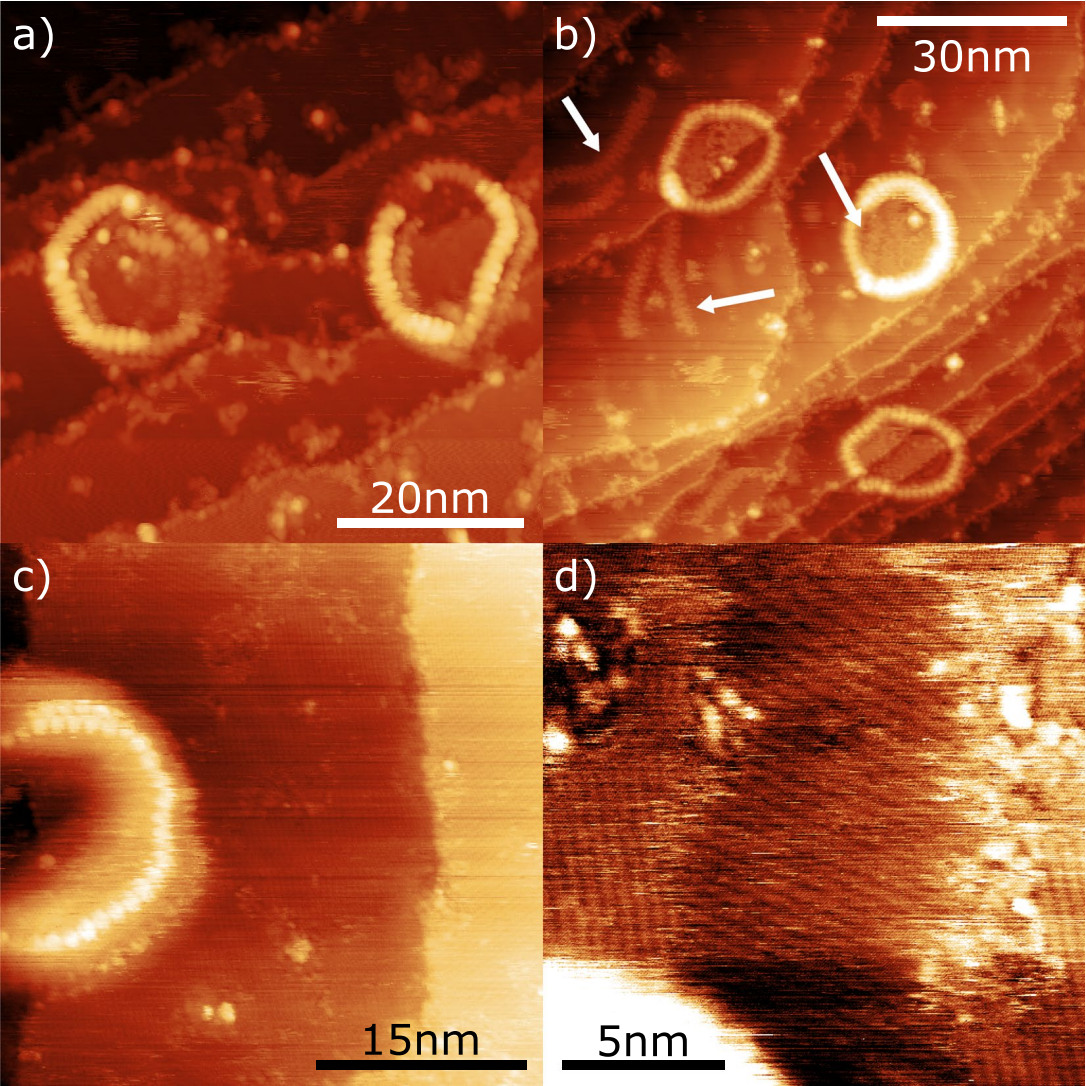
Details of the effect of elevated temperatures on the c-P40 rings in the presence of co-deposited TIPB. (**a**) STM image showing c-P40 rings after annealing the Au(111) surface to ~105 °C. Rings are broken in several places and rings within stacks have been laterally displaced (*I*_*set*_ = 30 pA, *V* = −1.8 V). (**b**) STM image of c-P40 rings on Au(111) after annealing to ~115 °C. Broken porphyrin chains inside and outside rings are indicated (white arrows), (*I*_*set*_ = 20 pA, *V* = −1.8 V). (**c**) STM overview image showing several large domains of co-deposited contaminant material on surface (*I*_*set*_ = 20 pA, *V* = −1.8 V). (**d**) Close up of area in (**c**) showing various domains of contaminant material (*I*_*set*_ = 20 pA, *V* = −1.8 V).

The methodology described above provides a promising route towards the application of macromolecular structures as templates for on-surface synthesis. It is, however, evident that there are also several technical limitations encountered as part of the current methodology which effect the formation, and characterisation, of extended TPB structures within the c-P40 rings. Experiments conducted to determine the thermal stability of the rings in the presence of TIPB revealed that c-P40 breaks at similar temperatures to those required to initiate the Ullmann-type coupling reaction (~110 °C). Heating samples to ~105 °C resulted in sections of the rings breaking, and further heating to ~120 °C caused almost all rings to rupture in at least one location. Examples of broken rings are shown in Fig. 3a,b (additional details are included within the Supplementary Information). Figure 3a shows two stacks of rings which have been affected by the anneal, with the topmost ring of the left stack exhibiting discontinuities in the observed contrast around the circumference of the ring (indicative of a break in the ring), and a section of the detached polymer material is observed within the pore of the ring. Similarly, the topmost porphyrin rings of the right stack exhibit discontinuities in contrast, but also appear to have been laterally displaced, with the upper layers of rings not resting upon the lower ones. Also commonly observed are linear chains of porphyrins, assigned to broken rings, seen both inside and outside intact rings (examples of these are highlighted by white arrows in Fig. 3b). These thermally decomposed structures create difficulties in both finding and imaging TPB structures on the surface (as mentioned previously for Fig. 2b). The weak intermolecular binding between the stacked rings (most likely mediated by *π*-*π* stacking) means that under standard tunnelling parameters (*I*_*set*_ = 20 pA, *V* = −1.8 V) it was observed that a spontaneous tip-sample interaction could result in the displacement or distortion of a stack of rings, preventing further imaging of that area.

STM characterisation of the surface following electrospray deposition of c-P40 reveals that a contaminant species has been co-deposited; found to be present over the majority of the surface, as shown in Fig. 3c, with such domains observed both inside and outside of the rings. A higher resolution image of this area (Fig. 3d) shows several domains of the contaminant arranged in rows oriented at 120° relative to each other; likely to be aligned to the major crystallographic directions of the surface lattice. The separation of these rows is found to be 0.51 ± 0.03 nm. Linear alkane chains are known to adsorb and form ordered domains similar to those observed here^42,43^, and we therefore propose that the contaminant consists of alkane chains either originating from the electrospray solvent, or potentially via decomposition of the c-P40 side groups. This contaminant is likely to have prevented TIPB adsorption in many areas (via a reduction in the sticking coefficient for TIPB incident upon the surface). In particular, the presence of the contaminant may act to passivate the surface, reducing the effectiveness of the surface as a catalyst. Overall, the co-deposition of the contaminant has the potential to limit the initial step of the Ullmann-type coupling process (C-I bond cleavage) required to form covalent bonds, reducing the efficiency of the on-surface reaction.

It is also worth noting a more general point with regards to obtaining STM images within the pore of the cyclic porphyrin rings; in such cases the sharpness of the STM probe (which can be approximated by the radius of curvature) may be comparable to the dimensions of the pore. Therefore, when imaging near the inside edge of c-P40 rings, the probe-surface separation and the separation between probe and the topmost ring within the stack may be of a similar magnitude - resulting in the measured tunnel current signal containing contributions, of similar magnitudes, from both the probe-surface and the probe- c-P40 ring interactions. Images produced this way will then be a convolution of both ring and surface states, resulting in the ‘blurring’ of the areas near the edges of rings. This effect is exacerbated for stacks containing several c-P40 rings, making the acquisition of images from an area inside a ring difficult. This issue can, in principle, be mitigated by preparing a high aspect-ratio STM probe. However, maintaining such a probe at room temperature (where diffusion of atoms at the apex of the probe is likely to be an important consideration) with a multi-component surface preparation (where diffusing molecular species may adsorb upon the probe) is particularly challenging.

Using a geometric model to simulate a probe imaging the surface within a pore (details included within Supplementary Information) we find that for stacks of rings consisting of three rings, combined with spherical probes of radius 15 nm or greater, it is expected that imaging inside a ring would lead to a considerable contribution to the measured tunnelling current from the interaction between the tip and the stack of rings, and hence STM images acquired in these regions will exhibit reduced resolution. This prediction supports our experimental observation that imaging molecular structures within the rings is non-trivial, and potentially explains the low contrast variation between the TPB networks and the surface, (see Fig. 2d) as the STM current signal is influenced by the probe-ring interaction.

## Conclusions

In conclusion, we have demonstrated the use of c-P40 porphyrin nanorings as a macromolecular template for the confinement of a reactant species which can subsequently participate in an Ullmann-type on-surface coupling reaction. Covalently bonded reaction products were observed both inside and outside of rings and these products were characterised by room temperature UHV-STM. The use of c-P40 as a template for confinement however is limited by a number of technical issues; most notably the low thermal stability of the rings, and the requirement for deposition of c-P40 via electrospray (introducing contaminant species to the surface), both of which limit the probability for the on-surface reactions to proceed. However, the results presented here demonstrate that large macromolecular structures can be used to template catalytically active surfaces, that on-surface reactions can be initiated within the pores of this templating structure, and that the reaction products can be observed within these templates.

## Supplementary information

Supplementary Information

## Data Availability

Supplementary information available on: Chemical structure of a potential reaction product formed within a porphyrin nanoring; Additional STM data for TIPB on Au(111), c-P40 on Au(111), and c-P40 with TPB structures; Details of the flattening factor; Details of Ag(111) nanoring deposition; Implications for STM resolution within porous structures; This material is available free of charge via the internet at: https://www.nature.com/srep/. The experimental data on which this work is based may be found at 10.17639/nott.6187.
