# Supplementary material for: On-Surface Synthesis within a Porphyrin Nanoring Template
Source: Sci Rep. 2019 Jun 27;9:9352. doi: 10.1038/s41598-019-45359-w (PMC6597552; doi:10.1038/s41598-019-45359-w)
Supplement: Supplementary file 1 — Supplementary Information [file 41598_2019_45359_MOESM1_ESM.pdf]

Supplementary Information
On-Surface Synthesis within a Porphyrin Nanoring Template

Chris J Judd[1], Dmitry V Kondratuk[2], Harry L Anderson[2], and Alex Saywell[1,*]

[1]School of Physics and Astronomy, The University of Nottingham, Nottingham, NG7 2RD, UK
[2]Department of Chemistry, Oxford University, Oxford, OX1 3TA, UK
[*]Correspondence and requests for materials should be addressed to A.S. email:Alex.Saywell@nottingham.ac.uk

1 Chemical structure of a potential TIPB reaction product formed within a porphyrin nanoring

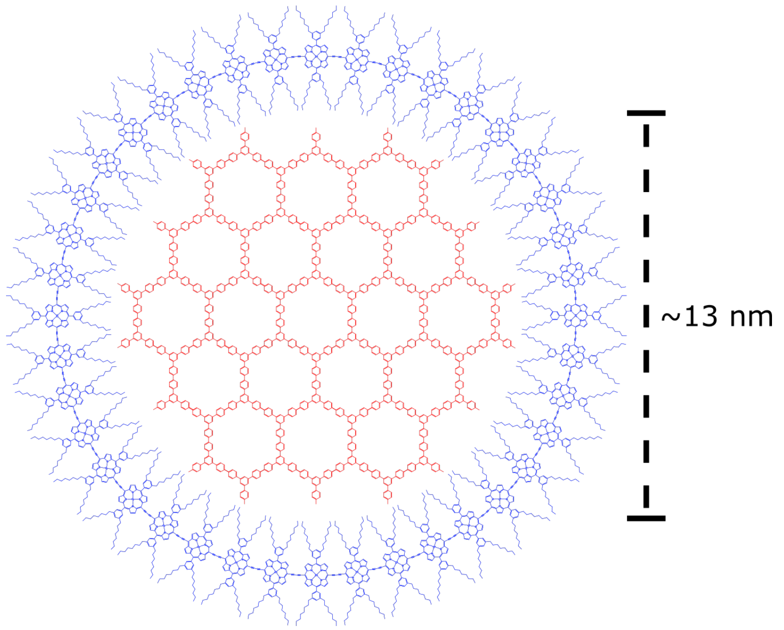

Figure S1: Scale model of a regular, 54 unit, 1,3,5-triphenylbenzene (TPB) covalently bonded structure which could conceivably fit within a c-P40 nanoring. TPB structure shown in red and c-p40 in blue.

2 Additional STM data for TIPB on Au(111), c-P40 on Au(111), and c-P40 with TPB structures

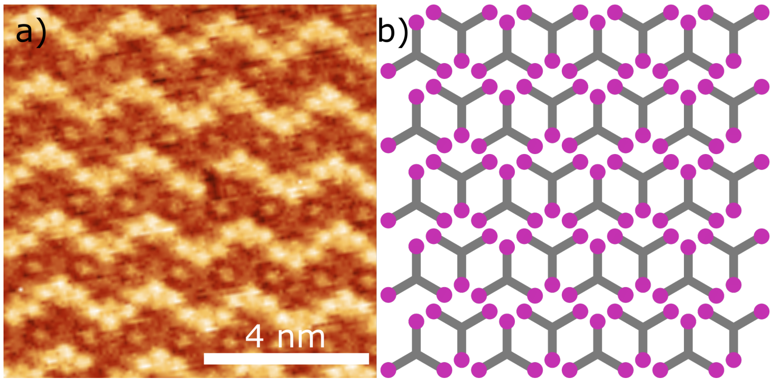

Figure S2: a) Scanning tunnelling microscopy (STM) image of 1,3,5-tris(4-iodophenyl)benzene (TIPB) structures observed on Au(111), prior to annealing. b) Structural model showing the regular zigzag arrangement formed by TIPB when deposited onto a Au(111) surface, prior to annealing. Purple circles represent iodine groups and grey lines the carbon backbone of the molecule. Structure has previously been reported [1]. Image parameters: $I_{set} = 50$ pA, $V = -0.8$ V.

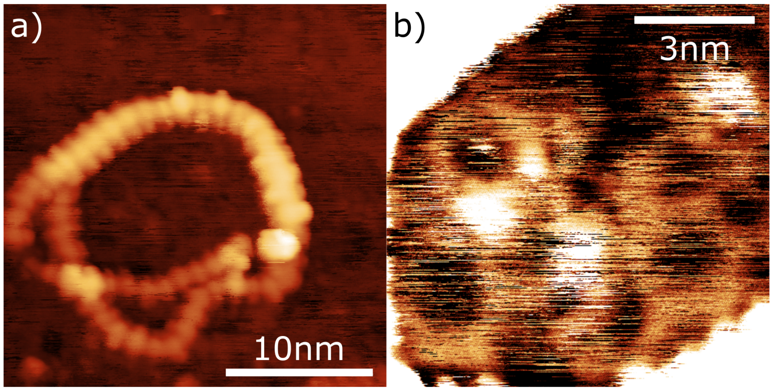

Figure S3: a) Duplicate of Figure 2d, from the accompanying manuscript, with an alternative contrast to show the position of the c-P40 rings more clearly (but with corresponding loss of contrast to the TPB structure within the ring). b) Duplicate of Figure 2e, from the accompanying manuscript, without the overlayed molecular structure - allowing the TPB structures to be viewed more clearly. Image parameters: $I_{set} = 20$ pA, $V = -1.8$ V.

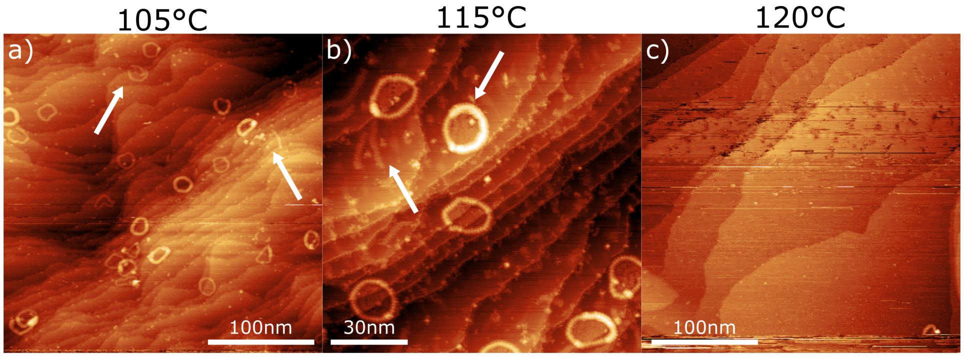

Figure S4: Example STM images acquired following annealing of a Au(111) surface patterned with c-P40 nanorings and co-deposited TIPB. White arrows highlight areas with broken rings. a) STM image acquired after an anneal of the surface at $\sim$ 105 °C for 30 mins. The majority of rings are observed to be intact, with a small number of broken chains present on the surface. b) STM image acquired after an anneal of the surface for 20 mins at $\sim$ 115 °C. A large numbers of broken nanorings are observed with some intact species present. c) STM image acquired after an anneal of the surface at $\sim$ 120 °C for 30 mins. The majority of rings on the surface are observed to break, with very little evidence for the presence of intact nanorings. Image parameters: $I_{set} = 30$ pA, $V = -1.8$ V.

3 Flattening Factor

The flattening factor, f, of an ellipse is a measure of compression of a circle; with a value of $f = 0$ corresponding to a perfect circle and a value of $f = 1$ a flat line. It is calculate by

$$f = \frac{a - b}{a}, \tag{1}$$

where a and b are the lengths of the major and minor axes of the ellipse respectively (see Figure S5a). The flattening factor for nanorings in this experiment was determined by measuring the lengths of the major and minor axes and was found to be $f = 0.31 \pm 0.03$ from 61 measurements. An example of this is shown in Figure S5b.

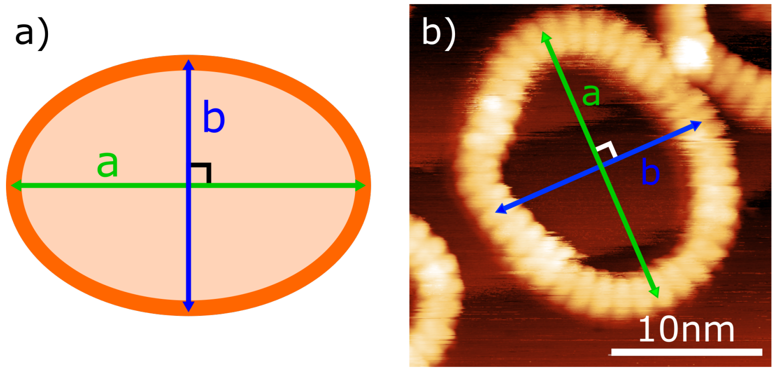

Figure S5: a) Diagram showing major (a) and minor (b) axes of an ellipse. b) Example image showing how measurements were made to calculate values for major and minor axes for nanorings. Image parameters: $I_{set} = 10$ pA, $V = -1.8$ V.

4 Details of Ag(111) nanoring deposition

Additional preliminary experiments were performed depositing c-P40 nanorings onto clean Ag(111) surfaces and an example image of this is shown in Figure 1f in the accompanying manuscript. Depositions were performed, using a solution of c-P40 in toluene and methanol (3:1 mixture), onto a sample held at 10^{-5} mbar, using a 2 μL/hour flow rate,for 15 minutes - applying a potential difference to the spray capillary of 2.5 kV .

5 Implications for STM resolution within porous structures

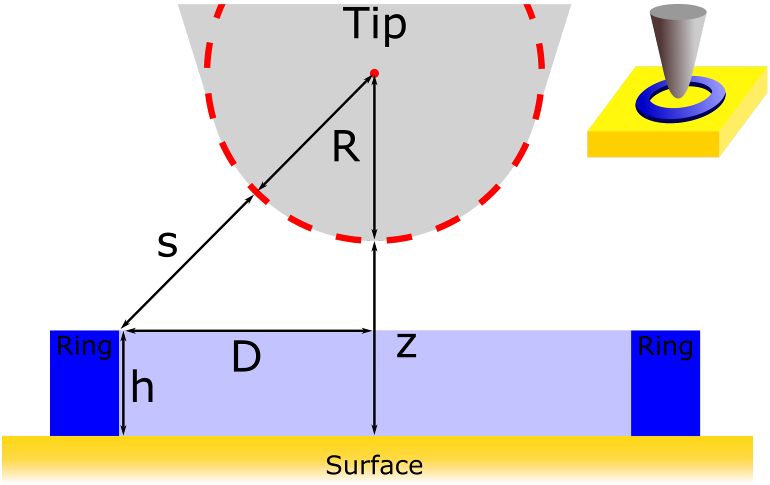

Figure S6: Schematic of tip-surface structure within a circular pore. Inset shows three dimensional perspective of the model.

The effect of obtaining STM images within a pore with appreciable vertical height, upon the resolution of the images obtainable, can be explored through a simple model (shown in Figure S6). The stack of rings is modelled as a hollow cylinder of height h. The STM tip can be considered a sphere of radius R, positioned a distance D away from the ring-stack and a height z above the surface. The distance s is the shortest separation between the edge of the ring-stack and any part of the tip. Using this model, it can be shown that

$$(s + R)^2 = D^2 + (z + R - h)^2, \tag{2}$$

and this can be rearranged to give

$$s - z = \sqrt{D^2 + (z + R - h)^2} - R - z. \tag{3}$$

When the tunnel current originating from the ring-stack (tunnelling through distance s) becomes comparable to the tunnel current between tip and surface, the presence of the ring-stack will have an appreciable effect upon the topography of the images acquired. Due to the sensitivity of STM

measurements with respect to the tip-surface separation (tunnel currents changing by an order of magnitude for a 1Å change in height) effects will be seen in images when $s \approx z$. The closest the tip can get to the ring stack ($D = D_{min}$) therefore occurs when $s - z = 0$. Using this, equation 3 can be rewritten

$$D_{min} = \sqrt{h(2R + 2Z - h)}. \tag{4}$$

Figure S8a presents data showing the variation of D_{min} with tip radius R at various ring heights and tip-surface separations. In this graph h was chosen to be 0.4, 0.7, and 1.0 nm, corresponding to the heights of 2, 3 or 4 nanorings respectively [2] and z was chosen to be 0.1, 0.5, and 1.0 nm above the surface. From this graph the role of several factors can be determined. Firstly, variation in tip-height has very little effect on tip-ring interactions compared to other factors. An increase in tip radius however results in large increase in D_{min}. This is as expected as a blunter tip (larger radius of curvature) will produce less clear images with lower resolution. The height of rings also has a significant impact on D_{min}, such that tip-ring interactions occur at smaller distances for shorter ring-stacks meaning that the STM tip can image closer to the inner edge of the ring for smaller stack-heights. Overall it can be seen that for all tip sizes and ring heights, images of areas taken within at least 1 nm of a ring will be affected by tip-ring interactions and this distance is significantly increased for higher ring-stacks and blunter tips.

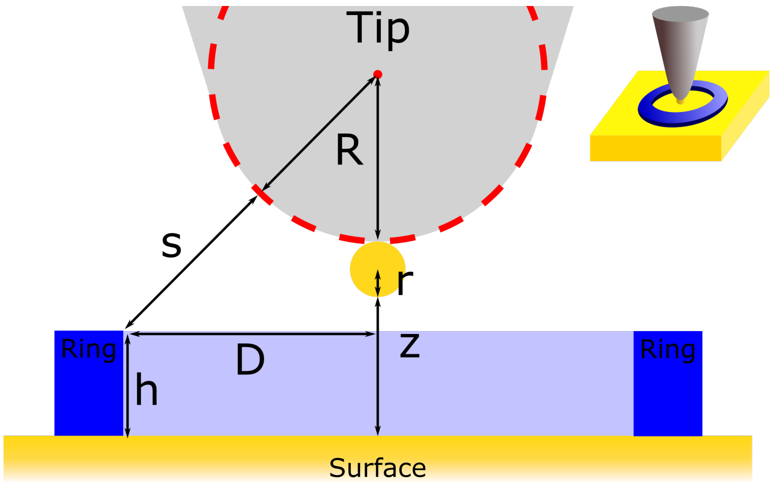

Figure S7: Extension of the model to include a tip terminated with a single Au atom.

This model can be extended by considering a tip terminated with a single Au atom of radius $r = 0.144$ nm, shown in Figure S7. Following the derivation steps above, it is found that

$$s - z = \sqrt{D^2 + (z + R + 2r - h)^2} - R - z, \tag{5}$$

and under the conditions of $s - z = 0$ at $D = D_{min}$

$$D_{min} = \sqrt{h(2R + 2Z - h) - 4r(Z + R + r - h)}. \tag{6}$$

This expression is similar to equation 4 but with an additional factor within the square root. This factor results in a reduction in D_{min} for all h, R and z values; as is shown in Figure S8b. The relationship shown in the graph is similar to the case with no atom on the tip (Figure S8a) but with all values of D_{min} reduced. This is as expected as tips terminated with single atomic species often produce images with enhanced resolution. This result is significant however as it shows that images can be taken from 0.5 nm of the ring edge without significant contributions from tip-nanoring interactions. This does however require very sharp tips, which can be very difficult to prepare under room temperature conditions for multi-component molecule-substrate systems. Such conditions further increase the difficulty of imaging structures within the rings, as seen in the experiments reported upon in the main manuscript. Based on the model outline here it is likely

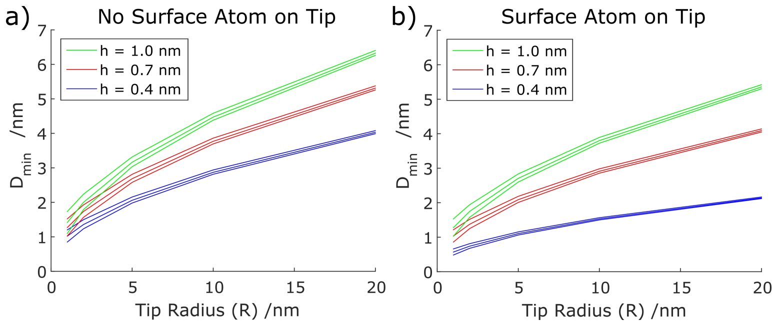

Figure S8: Graphs showing variation in the closest distance (D_{min}) the tip can approach a ring of height h before tip-ring interactions become comparable to tip-surface interactions. a) shows variation for a regular metallic tip of radius R and b) shows variation for a tip of radius R, terminated in a Au atom of radius $r = 0.144$ nm. In each graph, variation is shown for ring heights of 0.4, 0.7 and 1.0 nm, corresponding to stacks of two, three and four nanorings respectively. Tip heights, z, above the surface of 0.1, 0.5 and 1.0 nm are also shown for each h value, with lower z values producing lower values of D_{min}.

that for "blunt" tips, with $R \approx 15$ nm, the area inside three- or four-unit high stacks of rings will be effected by significant convolution between tip-surface and tip-ring interactions, and hence STM images acquired in these regions will exhibit reduced resolution.

References

[1] David Peyrot and Fabien Silly. On-Surface Synthesis of Two-Dimensional Covalent Organic Structures versus Halogen-Bonded Self-Assembly: Competing Formation of Organic Nanoarchitectures. *ACS Nano*, 10(5):5490–5498, May 2016.

[2] Dmitry V. Kondratuk, Luís M. A. Perdigão, Ayad M. S. Esmail, James N. O'Shea, Peter H. Beton, and Harry L. Anderson. Supramolecular nesting of cyclic polymers. *Nature Chemistry*, 7(4):317–322, April 2015.
